# Structure prediction of polyglutamine disease proteins: comparison of methods

**DOI:** 10.1186/1471-2105-15-S7-S11

**Published:** 2014-05-28

**Authors:** Jingran Wen, Daniel R Scoles, Julio C Facelli

**Affiliations:** 1 Department of Biomedical Informatics, University of Utah, Salt Lake City, Utah, USA; 2 Department of Neurology, University of Utah, Salt Lake City, Utah, USA

## Abstract

**Background:**

The expansion of polyglutamine (poly-Q) repeats in several unrelated proteins is associated with at least ten neurodegenerative diseases. The length of the poly-Q regions plays an important role in the progression of the diseases. The number of glutamines (Q) is inversely related to the onset age of these polyglutamine diseases, and the expansion of poly-Q repeats has been associated with protein misfolding. However, very little is known about the structural changes induced by the expansion of the repeats. Computational methods can provide an alternative to determine the structure of these poly-Q proteins, but it is important to evaluate their performance before large scale prediction work is done.

**Results:**

In this paper, two popular protein structure prediction programs, I-TASSER and Rosetta, have been used to predict the structure of the N-terminal fragment of a protein associated with Huntington's disease with 17 glutamines. Results show that both programs have the ability to find the native structures, but I-TASSER performs better for the overall task.

**Conclusions:**

Both I-TASSER and Rosetta can be used for structure prediction of proteins with poly-Q repeats. Knowledge of poly-Q structure may significantly contribute to development of therapeutic strategies for poly-Q diseases.

## Background

Knowledge of protein structure can be critical for devising therapeutic strategies for diseases in which protein dysfunction contributes to pathogenesis. For the polyglutamine (poly-Q) diseases, pathogenic poly-Q expansions typically cause gains of toxic functions associated with protein misfolding or aberrant interactions with RNAs or other proteins [1]. At least ten neurodegenerative disorders are caused by poly-Q expansions, including Huntington's disease (HD), dentatorubral and pallidoluysian atrophy (DRPLA), spinal and bulbar muscular atrophy (SBMA), and the poly-Q spinocerebellar ataxias [2] (SCA1, SCA2, SCA3, SCA6, SCA7, SCA8, and SCA17) [3-5]. The proteins involved in these diseases have no significant sequence, compositional or structural homologies [6,7] and numerous studies and observations have established that the length of the polyglutamine repeats plays a critical role in the progress and pathogenesis of these diseases [5,8]. Analysis from patients' data reveals that the expansion of polyglutamine repeats beyond certain pathological threshold causes the disease phenotype (Table 1) [9-12]. Also the number of the glutamines in the polyglutamine region is inversely correlated with age of onset [9,13-17]. For instance for SCA2, people with 32 or 33 repeats tend to first experience symptoms of SCA2 in late adulthood, while people with more than 45 repeats usually have symptoms by their teens [2].

**Table 1:** Length of poly-Qs in polyglutamine diseases.

	Gene	Wild-type allele repeat number	Mutant allele repeat number
SCA1	ATXN1	6-39	41-83
SCA2	ATXN2	13-31	> = 32
SCA3	ATXN3	12-43	60-89
SCA6	CACNA1A	<18	20-33
SCA7	ATXN7	<19	36-460
SCA8	ATXN8	15-50^a^	80-250^a^
SCA17	TBP	25-42	49-66
HD	HTT	10-26	>40
DRPLA	ATN1	6-35	>48
SBMA	AR	< =36	>38

**^a ^**The non-coding gene ATXN8OS gene may contribute to SCA8 pathogenesis. The number of CAG repeats in normal and mutant ATXN8 are assumed to be the same as the number of CTG repeats in normal and mutant ATXN8OS, as in [4].

One possible mechanism for these diseases pathology is the assembly of unfolded protein monomers into β-sheet amyloid fibers [18]. Both *in vivo *and *in vitro *studies have shown that the poly-Q expansion may lead to protein misfolding [19] and may cause a structure transition to form parallel β-helix and β-sheet folds [20]. Protein misfolding and aggregation has been shown to depend on the poly-Q length and the concentration of the protein [21-23]. As shown in [24] the poly-Q tract will form β-sheet structures when the number of the Qs increases resulting in an increase of the chance of aggregation. Therefore the understanding of the effect of the lengthening of the poly-Q repeat segment on protein folding can provide new insights and perhaps therapies for these diseases.

Although the association of the lengthening of the poly-Q repeats with the related polyglutamine diseases has been known for almost 20 years [25,26], high-resolution structural analysis of these proteins in their native context has eluded researchers [27] and only very limited experimental information exists. Kim has crystallized multiple structures of the N-terminal segment of huntingtin protein with 17 and 36 glutamines repeats [28,29], finding that the poly-Q regions exhibit conformational flexibility with α-helix, random coil, and extended loops [28,29]. These structures are the only crystal structures of poly-Q segments available in the RCSB PDB database. Computational modeling can provide valuable insights to this problem [23,30,31], but to our knowledge no comprehensive studies have been reported comparing the 3D structures predicted for these segments with the limited experimental data available.

The accuracy of the structures obtained using 3D structure prediction programs is improving rapidly, and some of the commonly available programs have shown excellent performance in the CASP competition [32]. However, all the 3D structure prediction programs are trained with a variety of proteins and their performance is usually evaluated on a general dataset [33]. There is no literature evidence reporting the performance of these programs on proteins containing poly-Q tracts. So it is necessary for us to evaluate the performance of these programs before we use them to predict the structure of polyglutamine disease proteins at large scale.

In this paper we present our results of the evaluation of the prediction performance of two efficient and popular 3D structure prediction programs, I-TASSER and Rosetta, on the N-terminal end of huntingtin protein with 17 glutamines (HTT17Q-EX1).

## Results

### Predicted models

As evidence shows that the poly-Q region can adopt different structures [28,29] in the proteins of interest for poly-Q diseases, it is not appropriate to seek the 'best structure' of this region, but it is more appropriate to look for ensembles of structures (generated by multiple independent runs) which can show overall trends and represent the variety of structures observed by experimental methods.

**Figure 1:**
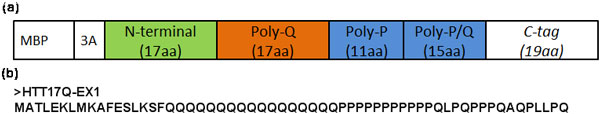
**The sequence construction of HTT17Q-EX1**. (a) sequence structure of the PDB records; (b) sequence used for structure prediction.

Following this reasoning, both Rosetta and I-TASSER were run 10 times using different random seeds for each run of 3D structure prediction of the HTT17Q-EX1 sequence shown in Figure 1(b). For each run we kept the five best models, so a total of 50 I-TASSER models and 50 Rosetta models were retained for analysis.

Each structure prediction program will return some parameters to estimate the accuracy of the models. For I-TASSER, the C-score, which lies in the (-5,2) range, is calculated for each model [34]. The C-scores of the best 50 I-TASSER models, listed in Table 2, range from -2.62 to -4.72.

**Table 2:** C-scores for the best I-TASSER models.

Model	# 1	# 2	# 3	# 4	# 5
Run 1	-2.91	-3.69	-3.33	-3.62	-4.72
Run 2	-2.84	-3.71	-3.31	-3.5	-4.42
Run 3	-2.81	-3.76	-3.48	-3.89	-3.74
Run 4	-2.62	-3.69	-3.32	-3.76	-3.49
Run 5	-3.02	-3.21	-3.91	-4.11	-3.42
Run 6	-2.67	-3.76	-3.48	-3.62	-4.37
Run 7	-2.77	-3.42	-3.96	-3.3	-3.51
Run 8	-3.09	-3.45	-4.09	-4.22	-4.27
Run 9	-2.73	-3.61	-3.38	-3.76	-4.42
Run 10	-2.62	-3.49	-3.75	-4.33	-4.01


The clustering algorithm from Rosetta was used to identify the most frequently sampled conformations. For each run we selected the five structures with the lowest energy from the structures encountered in the five different clusters in which the number of structures was greater than 10 on each. The energies of the total 50 Rosetta structures, listed in Table 3, they range from 16.06 to 20.13.

**Table 3:** Energy for the best Rosetta models.

Model	# 1	# 2	# 3	# 4	# 5
Run 1	16.061	16.349	18.609	19.656	19.956
Run 2	17.881	18.309	18.373	18.386	19.215
Run 3	16.943	17.598	17.639	18.306	19.436
Run 4	18.414	18.662	18.691	18.812	19.076
Run 5	16.74	18.004	18.192	18.3	19.015
Run 6	18.353	18.388	18.572	18.766	18.96
Run 7	17.435	18.897	19.571	19.603	19.617
Run 8	18.128	19.111	19.521	19.643	19.707
Run 9	17.317	17.586	17.655	17.916	18.69
Run 10	19.329	19.899	19.928	20.104	20.13

### Secondary structure

**Figure 2:**
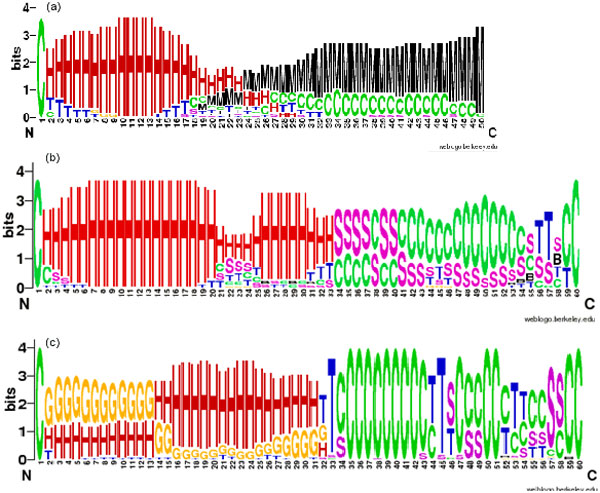
**Secondary structure WebLogo**. (a) PDB structures; (b) I-TASSER models; (c) Rosetta models. In (a) M represents the number of structures with missing values due to lack of resolution in the experimental data. The codes for secondary structure are as follows: H: α-helix; B: β-bridge; E: Strand; G: Helix-3; I: Helix-5; T: Turn; S: Bend; M: Missing data.

For better visualization, WebLogo [35] was used to display secondary structure patterns. The WebLogo of the secondary structures of the experimental PDB structures and the best I-TASSER and Rosetta models are shown in Figure 2. For easy description, we divided the sequence into three regions: the 17-residue head region including residues 1 to 17; the poly-Q region including residues 18 to 34 and C-terminal region including residues 35 to 60. As discussed in the original publication for the 21 PDB structures most crystals show α-helix in the head region, which is always well resolved, with only a few structures showing turns at the beginning and end of the head region. Both the I-TASSER and Rosetta best models reproduce the observed trends showing a majority of helix structures in the head region, but the I-TASSER structures show better agreement with the experimental findings showing a preference for α-helix, while the Rosetta structures show a mix of α-helix and 3-helix. The secondary structure, for the resolved structures, in the poly-Q region is more diverse showing a number of structures with α-helix, random coils and turns. The Pro-enriched C-terminal region is dominated, at least for the resolved structures in this region, by coil structures. Unfortunately, as depicted in Figure 2(a), the number of well resolved structures rapidly decreases beyond the head region making comparison with the experiments less reliable. None-the-less the overall experimental trends are reproduced by both I-TASSER and Rosetta, but it appears that the I-TASSER structures show more loops than the experimental data.

Overall I-TASSER appears to be superior reproducing quite well the stable α-helix structure of the N-terminal regions and showing increased diversity of structures in the poly-Q region and a predominance of coil structures in the C-terminal region.

### Reproducibility of I-TASSER and Rosetta results

In order to test the sensitivity of I-TASSER and Rosetta with the selection of the seeds used in the calculations, we have calculated the structure similarity using the TM-score between models obtained using the same prediction program. A total of 1225 TM-scores were generated comparing pairwise the best 50 I-TASSER and 50 Rosetta models, respectively.

**Figure 3:**
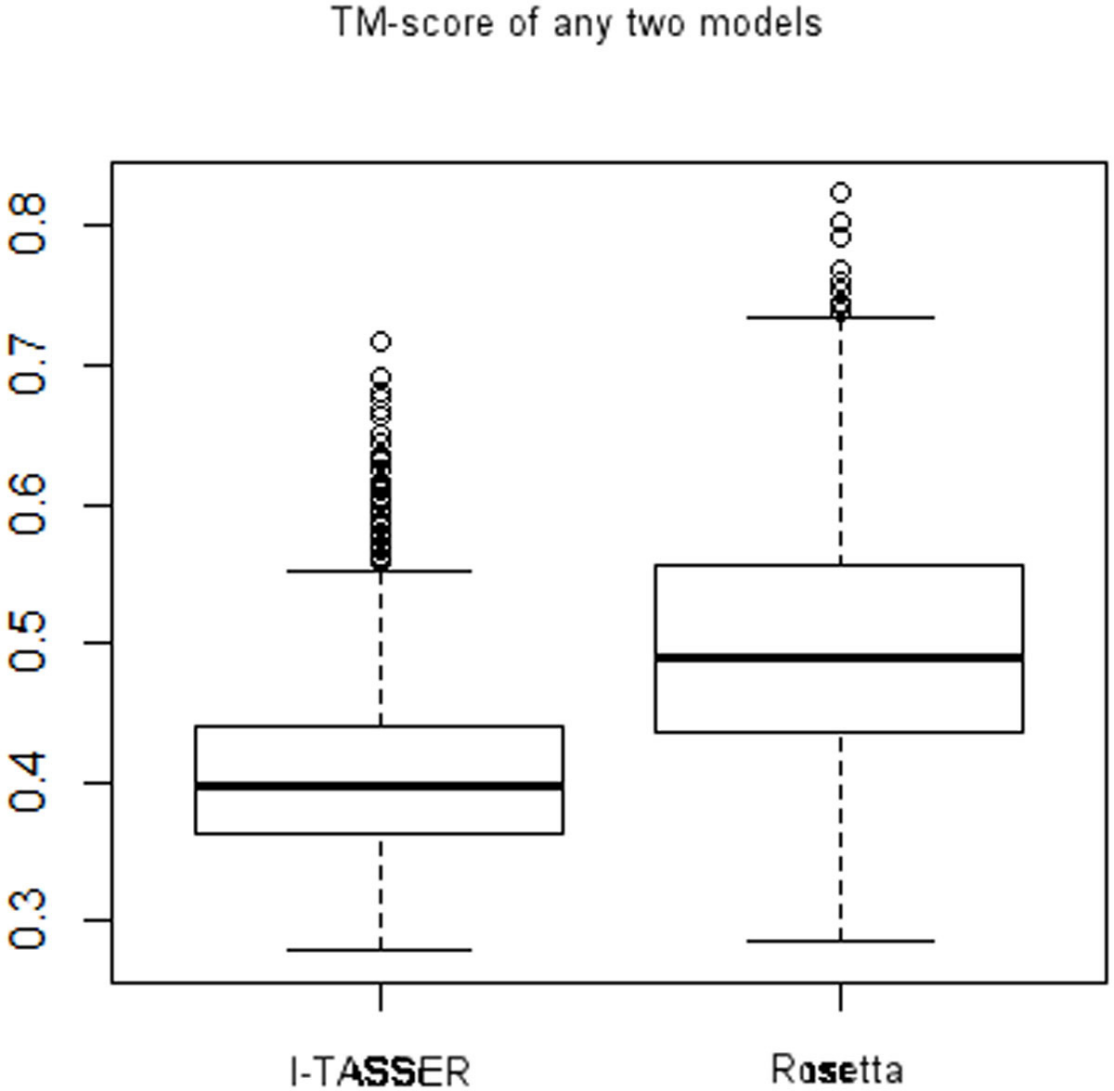
**Distribution of TM-scores of any two models from I-TASSER and Rosetta respectively**.

TM-scores between any two models from I-TASSER range from 0.2781 to 0.7163, with an average of 0.4086 and a standard deviation 0.0692. Whereas the TM-scores between any two Rosetta models range from 0.2865 to 0.8236, with an average of 0.4979 and a standard deviation 0.0892. The difference between TM-scores of I-TASSER and Rosetta is statistically significant (t-test, p < 0.001, Figure 3). The number of TM-scores greater than 0.5 is two times greater for Rosetta/Rosetta pairs than for I-TASSER/I-TASSER pairs, i.e. 561 pairs in Rosetta and 126 pairs in I-TASSER have scores larger than 0.5.

When comparing only the best models of each run, the TM-scores range from 0.4539 to 0.6813 for I-TASSER (Table 4) and from 0.2872 to 0.6879 for Rosetta (Table 5). Therefore the best models of each run from I-TASSER are more similar among themselves than those from Rosetta, i.e. 33 pairs of the 45 structure pairs have TM-scores greater than 0.5 for I-TASSER, whereas for Rosetta, only 18 pairs of best models have TM-scores greater than 0.5.

**Table 4:** TM-scores between the best models from I-TASSER.

run	# 2	# 3	# 4	# 5	# 6	# 7	# 8	# 9	# 10
# 1	0.48	0.48	0.63	0.48	0.54	0.57	0.49	0.51	0.46
# 2		0.56	0.54	0.55	0.48	0.54	0.45	0.58	0.46
# 3			0.54	0.63	0.53	0.51	0.54	0.56	0.51
# 4				0.50	0.52	0.66	0.49	0.56	0.49
# 5					0.60	0.51	0.58	0.59	0.54
# 6						0.51	0.68	0.54	0.49
# 7							0.50	0.57	0.45
# 8								0.50	0.52
# 9									0.50

**Table 5:** TM-scores between best models from Rosetta.

run	# 2	# 3	# 4	# 5	# 6	# 7	# 8	# 9	# 10
# 1	0.30	0.43	0.37	0.42	0.42	0.53	0.40	0.38	0.43
# 2		0.30	0.34	0.31	0.39	0.28	0.34	0.32	0.38
# 3			0.52	0.53	0.53	0.65	0.47	0.57	0.50
# 4				0.48	0.53	0.47	0.68	0.49	0.43
# 5					0.63	0.52	0.48	0.58	0.61
# 6						0.52	0.47	0.64	0.59
# 7							0.41	0.56	0.55
# 8								0.46	0.42
# 9									0.60

The sensitivity to the selected random seeds was also evaluated at the run level. TM-scores were calculated for the structures of any 5 models in one run compared with any 5 models of other runs. The number of pairs with TM-score greater than 0.5 between any two experiments is shown in Table 6 for I-TASSER and Table 7 for Rosetta. For I-TASSER, the number of pairs with TM-score greater than 0.5 ranges from 0 to 6. There are 6 pairs with TM-scores greater than 0.5 between Run 4 and Run 7, however, no pairs with TM-scores greater than 0.5 between Run 1 and Run 8. For Rosetta, the number of pairs with TM-score greater than 0.5 at run level ranges from 5 to 20. 20 of 25 pairs are with TM-scores greater than 0.5 between Run 3 and Run 7, which is the best. The smallest number of pairs for Rosetta is 5, which shows in 3 pairs, Run 1 and Run 6, Run 6 and Run 8, Run 5 and Run 8.

**Table 6:** Number of pairs with TM-score greater than 0.5 between any two runs of I-TASSER.

run	# 2	# 3	# 4	# 5	# 6	# 7	# 8	# 9	# 10
# 1	2	3	3	1	3	3	0	5	1
# 2		2	2	2	2	3	2	2	1
# 3			3	3	2	3	2	1	4
# 4				3	2	6	3	5	1
# 5					3	5	3	2	4
# 6						4	1	5	2
# 7							4	5	2
# 8								3	2
# 9									2

**Table 7:** Number of pairs with TM-score greater than 0.5 between any two runs of Rosetta.

run	# 1	# 2	# 3	# 4	# 5	# 6	# 7	# 8	# 9	# 10
# 1	4	6	10	11	8	5	11	12	8	9
# 2		2	11	8	7	6	13	11	11	10
# 3			6	15	9	13	20	16	18	16
# 4				3	9	7	14	13	16	14
# 5					4	8	15	5	16	15
# 6						1	8	5	13	9
# 7							5	15	16	14
# 8								8	7	13
# 9									9	17
#10										6

These results show that our ensemble approach to predict the structure of proteins associated with poly-Q diseases appears to be appropriate. Using multiple seeds it is possible to obtain an ensemble of structures that show reasonable diversity, but still retain the main features. We believe that this approach is quite promising because it can incorporate in future analysis the diverse structure of which appears to be an emerging observation from the limited experimental data on these proteins.

### Validity evaluation of I-TASSER and Rosetta

As depicted in Figure 2(a) not all of the 21 PDB structures have been resolved in the poly-Q region, which is our main interest. For instance, the longest well resolved poly-Q region is the B chain of the 3IOW [PDB: 3IOW] structure in which all the 17 Qs structures are resolved, whereas for the A chain of the 3IOT [PDB: 3IOT] structure only one Q has been resolved. Also, there are numerous gaps in several structures as some of the residues are not resolved. Taking this into account and in order to make an accurate comparison with the experimental ones in the region of interest, only PDB structures in which at least 9 (more than half the total number) of consecutive Qs in the poly-Q region show well resolved structures were used for the evaluation of the results produced with I-TASSER and Rosetta. There are ten PDB structures that meet this criteria: the B chain of 3IO4 [PDB: 3IO4] (3io4_b), the C chain of 3IO4 [PDB: 3IO4] (3io4_c), the B chain of 3IO6 [PDB: 3IO6] (3io6_b), the C chain of 3IO6 [PDB: 3IO6] (3io6_c), the C chain of 3IOR [PDB: 3IOR] (3ior_c), the B chain of 3IOT [PDB: 3IOT] (3iot_b), the C chain of 3IOU [PDB: 3IOU] (3iou_c), the B chain of 3IOV [PDB: 3IOV] (3iov_b), the C chain of 3IOV [PDB: 3IOV] (3iov_c), and the B chain of 3IOW [PDB: 3IOW] (3iow_b). The number of consecutive Qs in each structure is shown in Table 8.

**Table 8:** Numbers of Qs in the PDB structures.

PDB structure	Number of Qs
3io4_b	10
3io4_c	11
3io6_b	14
3io6_c	10
3ior_c	13
3iot_b	12
3iou_c	14
3iov_b	11
3iov_c	15
3iow_b	17

The best 50 I-TASSER and 50 Rosetta models were compared with these 10 PDB structures using the TM-align program. TM-scores, root-mean-square deviation (RMSD), aligned number of residues, sequence identity and the structure superposition were obtained from TM-align [36]; the number of exact matches and the number of exact matched Qs were extracted from the structure alignment and finally the exact structure overlap (ESO) and exact structure overlap of Qs (ESOP) were calculated using equation (2) and equation (3) given in the methods section. The values of each similarity parameter considered here are shown in Table 9 along with the p-values assessing the significance of the difference between the I-TASSER and Rosetta results.

**Table 9:** Distribution of structure superposition parameters between predicted models and PDB structures

	I-TASSER	Rosetta	p-value
TM-score	0.50 ± 0.06	0.45 ± 0.06	<0.0001
RMSD (Å)	1.53 ± 0.34	1.74 ± 0.34	<0.0001
Aligned number	24.05 ± 2.14	25.56 ± 2.41	<0.0001
Sequence Identity ^a^	(0.30,0.71)	(0.38,0.52)	<0.0001
Exact Match (<5.0 Å) ^a^	(0,16)	(0,0)	<0.0001
Exact Qs Match(<5.0 Å) ^a^	(0,1)	(0,0)	<0.0001
Total Qs Match(<5.0 Å) ^a^	(5,8)	(8,11)	<0.0001
Exact Match (other) ^a^	(0,0)	(0,0)	<0.0001
Exact Qs Match(other) ^a^	(0,0)	(0,0)	<0.0001
Total Qs Match(other) ^a^	(0,1)	(0,1)	<0.0001
Exact Match (all) ^a^	(6,25)	(0,0)	<0.0001
Exact Qs Match (all) ^a^	(0,1)	(0,0)	<0.0001
Total Qs Match (all) ^a^	(6,8)	(9,12)	<0.0001
ESOP ^a^	(0,9.09)	(0,0)	<0.0001
ESO ^a^	(0, 53.13)	(0,0)	<0.0001

^a ^The values between brackets represent the value of the property for the best structure superposition at the first and third quartile, respectively, of their distributions.

The average TM-score of I-TASSER/PDB superposition pairs is 0.50 and the average TM-score of Rosetta/PDB pairs is 0.45, reflecting the fact that 253, of the 500, I-TASSER/PDB pairs have TM-scores greater than 0.5 while only 87 pairs of the Rosetta/PDB pairs have TM-scores greater than 0.5. The average RMSD of I-TASSER/PDB pairs (1.53 Å) is also smaller than that of Rosetta/PDB pairs (1.74 Å). Other TM-align parameters depicted in Table 9 also show that I-TASSER performs better than Rosetta in this test.

The structure overlap scores, ESOP and ESO, for I-TASSER models are also better than those for Rosetta models. For instance more than 75% of the Rosetta models have no exact match in the poly-Q region nor for the entire sequence, whereas the 75% quantile of the ESO and ESOP scores for I-TASSER are 53.13 and 9.09, respectively. The statistical tests have shown that these differences are significant (Table 9).

Fifty of the I-TASSER/PDB structure superpositions have ESOP values greater than or equal to 50, which means that 50 pairs have more than 50% of Qs in the poly-Q region with exact match. These 50 pairs include 9 of the 10 PDB structures, so 9 of the 10 structures have corresponding I-TASSER models with very good matches in the poly-Q regions. In contrast only 5 of these 10 structures have corresponding Rosetta/PDB structure superposition matches when the same criteria are used.

**Figure 4:**
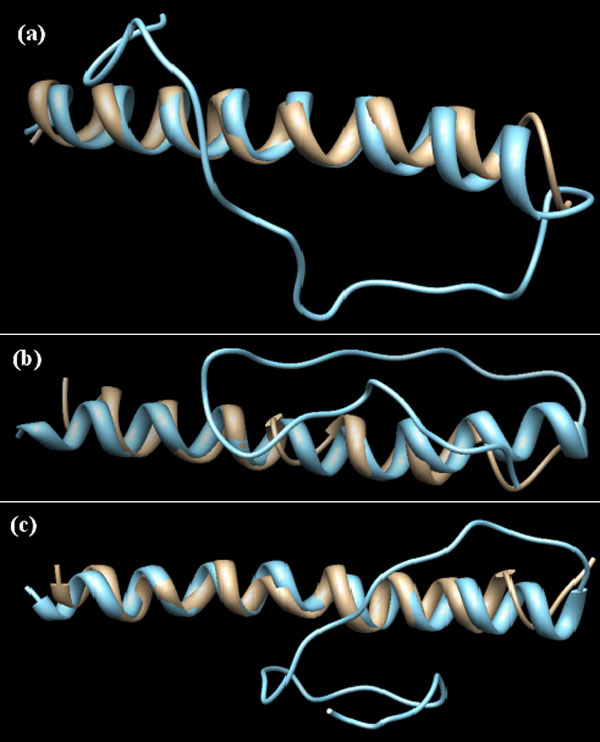
**Structure superposition of predicted models and PDB structures**. Structure superposition of predicted models and PDB structures with TM-score>0.5 and ESOP = 100. (a) I-TASSER third model of the tenth run with 3io6_b; (b) Rosetta forth model in first run with 3iou_c; (c) Rosetta third model in fifth run with 3iow_b. tan: PDB structure, sky-blue: predicted models. The N-terminal end of each structure is shown on the left.

The best matches between the predicted structures by I-TASSER and Rosetta, respectively, and one of the PDB structures considered here are depicted in Figure 4. The I-TASSER structure best match is with the B chain of 3IO6 [PDB: 3IO6]; the match has a TM-score of 0.56 and the ESOP score of 100. The best two matches for Rosetta structures show matches with the C chain of 3IOU [PDB: 3IOU] and the B chain of 3IOW [PDB: 3IOW]. Their TM-scores are 0.5074 and 0.5057, respectively, and the ESOP score of 100.

## Discussion

This study evaluated two software tools for predicting from amino acid sequences, the 3D structures of the poly-Q regions of proteins related to polyglutamine diseases. Pathogenic neurodegenerative poly-Q proteins were used as a model, for relevance to developing structure-specific therapeutics based on normal vs. poly-Q expanded protein structures. Two highly recognized and efficient 3D structure prediction programs, I-TASSER and Rosetta, were evaluated to assess their performance for structure prediction using segments of the huntingtin protein harboring poly-Q repeats. Both I-TASSER and Rosetta produced good results.

When tested for structure stability under changes of the initial random seed, Rosetta shows less variability than I-TASSER. This means that if we run Rosetta and I-TASSER several times respectively, it is possible that we will get less variance in the results from Rosetta than from I-TASSER. None-the-less, both programs produce a reasonable ensemble of structures with sufficient diversity and without extreme deviations. Several studies have illustrated that the poly-Q repeat regions of these proteins are highly disordered with structure flexibility [31], but this has not been quantified experimentally. Therefore it is challenging to discriminate among these two approaches using these criteria. In consequence we must conclude that both I-TASSER and Rosetta are suitable for the task on predicting ensemble structures of protein containing poly-Q segments.

The accuracy of the prediction program is a very important factor that we evaluated here. In this study, the structure similarity between the predicted models and the PDB experiment structures available was used to evaluate the validity of the prediction programs. The root-mean-square deviation (RMSD) score is the most often-used parameter to calculate the structure similarity, but a drawback of its use is that a relatively small local variation can result in a high RMSD [37]. TM-score weights the close atom pairs stronger than the distant matches, and it is more sensitive to the topology fold than the RMSD [37]. Besides the global similarity measured by TM-score, more restricted scores on the exact match of two structures were also calculated. The exact structure overlap (ESO), derived from the structure overlap (SO) score [38], was introduced and instead of calculating the number of aligned pairs it counts the exact match pairs, which not only counts aligned residues but also residues that lie in the same positions in both the sequences of predicted model and PDB structure. The exact structure overlap of poly-Q repeat (ESOP) is the special version of ESO, which is used to measure the prediction accuracy in the poly-Q region. Considering the TM-score, ESO and ESOP together gives a more comprehensive view of similarity between the predicted model and the PDB experimental structures from both a global and a local aspect. The ESO score and ESOP score can be used for similarity comparison tasks, especially if there are regions which play more critical roles than others.

Rosetta models have a larger number of aligned residues on average than I-TASSER, but the average RMSD values and TM-scores are much higher (lower) than that of I-TASSER. So when the Rosetta models are aligned with the PDB structures, the distance between the models and the experimental structures is large, which is not a good sign for good structural matches. On the contrary, I-TASSER models aligned better with PDB structures not only with better RMSD and TM-scores, but also better ESO and ESOP scores. This can also be seen from the secondary structures patterns. When considering specific structure pairs, both I-TASSER and Rosetta have predicted models which can match the PDB structures with good global (TM>0.5) and local (ESO>=50 and ESOP>=50) structures. So both Rosetta and I-TASSER have the ability to get the native models, but for the overall performance, I-TASSER appears to be better than Rosetta.

As several models are returned by the structure prediction programs, it is important to have criteria to select the best models. However, the model with the lowest energy in the prediction program may not be the best model for reproducing the poly-Q regions. For instance for Rosetta, the two predicted models with TM-score greater than 0.5 and ESOP of 100 (Figure 4(b) and 4(c)) are not the models with the lowest energy in that Rosetta run. This is true also for the I-TASSER model with TM-score greater than 0.5 and ESOP of 100 (Figure 4(a)). In fact, of the 29 good models which have TM-score greater than 0.5 and ESOP score greater than 50, only one model is ranked as the best by I-TASSER.

## Conclusions

Both I-TASSER and Rosetta can be used for *in silico *studies of the structures of proteins with poly-Q repeats related to neurodegenerative diseases. However, I-TASSER shows better performance than Rosetta when considering the overall agreement between results produced using these two prediction models with the limited experimental results available for comparison.

Both I-TASSER and Rosetta are computationally efficient as both applications can be easily parallelized by executing numerous jobs each with a unique random seed.

In our future studies we will attempt to predict the change of the structure as function of the number of Qs in the poly-Q repeat segment for all the proteins involved in poly-Q neurological diseases. Ideally we could use both these two programs to predict structures of the poly-Q disease related proteins. This could provide a quasi "crowdsourcing" mechanism to cross check the results, but may prove computationally too expensive (see Methods). Therefore the results presented here suggest that studies should be, at least initially, performed using I-TASSER.

## Methods

### Poly-Q segments

We searched the RCSB PDB database [39] for structures with more than 10 consecutive glutamines in their sequences on November 2012. A total of 11 structures were retrieved, including 7 of the first exon of the huntingtin protein with 17 glutamines (HTT17Q-EX1) [28] and 4 of the first exon of huntingtin protein with 36 glutamines (HTT36Q-EX1) [29]. Figure 1(a) shows the sequence construction for the X-Ray diffraction experiment on HTT17Q-EX1 which was expressed and crystallized as a maltose-binding (MDP) fusion protein [28]. The same methods were used to get the crystal structure of HTT36Q-EX1, but the resolution of the HTT36Q-EX1 is of such poor quality that only HTT17Q-EX1 structures were used in this study.

PDB identification numbers of the 7 HTT17Q-EX1 crystal structures used here are 3IO4 [PDB: 3IO4], 3IO6 [PDB: 3IO6], 3IOT [PDB: 3IOT], 3IOU [PDB: 3IOU], 3IOR [PDB: 3IOR], 3IOV [PDB: 3IOV] and 3IOW [PDB: 3IOW]. Each crystal includes a trimer of MDP-HTT17Q-EX1, so a total of 21 structures of HTT17Q-EX1 were considered. Figure 1(b) shows the sequence of the HTT17Q-EX1 used as the input of the 3D structure prediction.

### Protein 3D structure prediction

Two protein structure prediction programs were used in this study, I-TASSER and Rosetta. Both I-TASSER and Rosetta have been used by thousands users and they are among the few programs which can handle large proteins with more than 1000 residues [34,40].

I-TASSER is the 3D structure prediction program based on multiple-threading alignments and iterative template fragment assembly simulations [41]. I-TASSER is a fully automated method and was used without further modifications, but we have verified that none of the templates corresponding to the structures 3IO4 [PDB: 3IO4], 3IO6 [PDB: 3IO6], 3IOT [PDB: 3IOT], 3IOU [PDB: 3IOU], 3IOR [PDB: 3IOR], 3IOV [PDB: 3IOV] and 3IOW [PDB: 3IOW] was included in the knowledge data used in the version of I-TASSER used here. Rosetta is a flexible software suite for macromolecular modeling, which includes tools for structure prediction and design [42]. Rosetta *ab initio *module was used in this study. For Rosetta, the quota protocol fragment picking was used to generate 3-mers and 9-mers fragments, which took into account the secondary structure predictions by PsiPred [43], Jufo9D Server [44] and SAM-T08 [45] as the quota pools. The weight given to the each quota pool was assigned following reference [46] and 200 fragments were picked from the total of 700 candidates available from both 3-mers and 9-mers fragments. The default parameters were used for Rosetta *ab initio *modelling with the number of output structures set as 5000, the default parameters also were used for Rosetta cluster module.

We installed I-TASSER Version 2.1 and Rosetta Version 3.4 in a cluster at the Center for High Performance Computing (CHPC) of University of Utah, where all computations were performed. As a fully automated program, the number of decoys to screen and the number of simulation jobs in I-TASSER are fixed, whereas Rosetta is much more flexible and users can define the output number of structures and the number of parallel simulation jobs, making it much more adaptable to the hardware architecture used. So it is difficult to compare the computational cost of the two programs. However, for the modelling tasks with the parameters used in our simulation, the total CPU time for I-TASSER to finish one HTT17Q-EX1 (60 amino acid residues) prediction was, in average, 24.58 hours using one core in a 2.4 GHz dual-core Opteron processor, whereas the average total CPU time for Rosetta to finish one HTT17Q-EX1 prediction with 5000 prediction structures was about 50.91 hours in the same computing environment.

### 3D structure alignment

To assess 3D structure similarity, TM-align was used for structure comparison and alignment [36]. The TM-score calculated by TM-align, which lies in (0,1] interval, is considered a good measure of the similarity of two structures [37]. A TM-score of less than 0.17 indicates a random alignment, whereas TM-score greater than 0.5 indicates that the two structures are generally in the same fold [37].

### Similarity measurement

Besides the TM-score, exact structure overlap (ESO) and exact structure overlap of poly-Qs (ESOP) were also used to measure the similarity of two structures. The words 'exact' here means the aligned residues are within certain threshold, 5Å in this study, and that they are the same residue in the HTT17Q-EX1 sequence. For example, if a serine (SER) in the 16th position of the predicted structure of HTT17Q-EX1is aligned, within the distance threshold, with the serine (SER) in the 16th position of PDB experimental structure, the 16SER-16SER is an exact match. ESO and ESOP is derived from the Structure Overlap (SO) which is a standardized score to compare the structure alignments and measure the local similarity of two structures [38]. The SO score is calculated as:

(1) $$SO=100\times \frac{L\left(A\right)}{\text{min}\left(Lm,Le\right)}$$

where *L(A) *is the structure alignment length; the *Lm *and *Le *are the length of the predicted model and the experimental structure, respectively.

We have modified Equation (1) to meet the aim of more strict structure comparison, and get the ESO score:

(2) $$ESO=100\times \frac{L\left(EA\right)}{\text{min}\left(Lm,Le\right)}$$

where *L(EA) *is the length of exact match; *Lm *and *Le *is the length of predicted model and the length of the PDB experimental structure respectively.

The structure of poly-Q region may play a more important role than other positions. In this study, the ESOP score is calculated to evaluate the structure similarity of the poly-Q regions. The ESOP is a special version of ESO, and it is calculated as:

(3) $$ESOP=100\times \frac{L\left(EAQ\right)}{\text{min}\left(LQm,LQe\right)}$$

where *L(EAQ) *is the length of the exact match of Qs; *LQm *and *LQe *are the length of poly-Q in predicted model and PDB experimental structure respectively.

### Secondary structure calculation

The secondary structure of the predicted models and the PDB experimental structures were calculated using the DSSP algorithm, which is an algorithm to standardize secondary structure assignment [47]. Secondary structures assigned by DSSP are 8 conformational states, including α-helix, β-bridge, strand, 3-helix, 5-helix, turn, bend, and random coil.

The results of DSSP are the secondary structures represented by one letter for each position. In order to get a better view of the results, 'WebLogo 3 ' [35] was used to plot the secondary structure logo at each position. The overall height of the stack indicates the secondary structure conservation at that position, and the height of the symbols within the stack indicates the relative frequency of each secondary structure type at that position.

### 3D structure visualization

The 3D structure and the 3D structure superposition were visualized in the UCSF Chimera software, a free program for molecular graphics and analysis [48].

### Statistics

To depict the data distribution of the parameters calculated here, the (mean value ± standard deviation) is listed for data with normal distribution, whereas for data that do not follow the normal distribution, the 25% quantile and 75% quantile values are listed.

The Student t test was applied for data with normal distribution and the Wilcoxon ranked test was performed on other data sets to assess significance. The significant level was set at 0.05. All the statistic work was done in the R environment which is a free software environment for statistical computing and graphics [49].

## Competing interests

The authors declare that they have no competing interests.

## Authors' contributions

JCF and JW designed the study; JW did the research work; JW, DRS and JCF discussed the results within the clinical and biochemical framework. All authors read and approved the final manuscript.

## Declarations

The cost for the publication of the article has been funded by the corresponding author.

This article has been published as part of *BMC Bioinformatics *Volume 15 Supplement 7, 2014: Selected articles from the 10th Annual Biotechnology and Bioinformatics Symposium (BIOT 2013). The full contents of the supplement are available online at http://www.biomedcentral.com/bmcbioinformatics/supplements/15/S7

## References

[B1] Wetzel R Physical chemistry of polyglutamine: intriguing tales of a monotonous sequence Journal of molecular biology 2012 421 4-5 466 490 10.1016/j.jmb.2012.01.030 22306404 PMC3362671

[B2] Matilla-Duenas A Corral-Juan M Volpini V Sanchez I The spinocerebellar ataxias: clinical aspects and molecular genetics Advances in experimental medicine and biology 2012 724 351 374 10.1007/978-1-4614-0653-2_27 22411256

[B3] Zoghbi HY Orr HT Glutamine repeats and neurodegeneration Annual review of neuroscience 2000 23 217 247 10.1146/annurev.neuro.23.1.217 10845064

[B4] Moseley ML Zu T Ikeda Y Gao W Mosemiller AK Daughters RS Chen G Weatherspoon MR Clark HB Ebner TJ Bidirectional expression of CUG and CAG expansion transcripts and intranuclear polyglutamine inclusions in spinocerebellar ataxia type 8 Nature genetics 2006 38 7 758 769 10.1038/ng1827 16804541

[B5] Michalik A Van Broeckhoven C Pathogenesis of polyglutamine disorders: aggregation revisited Human molecular genetics 2003 12 Spec No 2 R173 186 14504263 10.1093/hmg/ddg295

[B6] Albrecht M Golatta M Wullner U Lengauer T Structural and functional analysis of ataxin-2 and ataxin-3 European journal of biochemistry / FEBS 2004 271 15 3155 3170 10.1111/j.1432-1033.2004.04245.x 15265035

[B7] Williams AJ Paulson HL Polyglutamine neurodegeneration: protein misfolding revisited Trends in neurosciences 2008 31 10 521 528 10.1016/j.tins.2008.07.004 18778858 PMC2580745

[B8] Magana JJ Velazquez-Perez L Cisneros B Spinocerebellar ataxia type 2: clinical presentation, molecular mechanisms, and therapeutic perspectives Molecular neurobiology 2013 47 1 90 104 10.1007/s12035-012-8348-8 22996397

[B9] Walters RH Murphy RM Examining polyglutamine peptide length: a connection between collapsed conformations and increased aggregation Journal of molecular biology 2009 393 4 978 992 10.1016/j.jmb.2009.08.034 19699209 PMC2764006

[B10] Garden GA La Spada AR Molecular pathogenesis and cellular pathology of spinocerebellar ataxia type 7 neurodegeneration Cerebellum (London, England) 2008 7 2 138 149 10.1007/s12311-008-0027-y PMC4195584 18418675

[B11] Costa Mdo C Paulson HL Toward understanding Machado-Joseph disease Progress in neurobiology 2012 97 2 239 257 10.1016/j.pneurobio.2011.11.006 22133674 PMC3306771

[B12] Imarisio S Carmichael J Korolchuk V Chen CW Saiki S Rose C Krishna G Davies JE Ttofi E Underwood BR Huntington's disease: from pathology and genetics to potential therapies The Biochemical journal 2008 412 2 191 209 10.1042/BJ20071619 18466116

[B13] Pulst SM Nechiporuk A Nechiporuk T Gispert S Chen XN Lopes-Cendes I Pearlman S Starkman S Orozco-Diaz G Lunkes A Moderate expansion of a normally biallelic trinucleotide repeat in spinocerebellar ataxia type 2 Nature genetics 1996 14 3 269 276 10.1038/ng1196-269 8896555

[B14] Zoghbi HY Jodice C Sandkuijl LA Kwiatkowski TJ McCall AE Huntoon SA Lulli P Spadaro M Litt M Cann HM The gene for autosomal dominant spinocerebellar ataxia (SCA1) maps telomeric to the HLA complex and is closely linked to the D6S89 locus in three large kindreds American journal of human genetics 1991 49 1 23 30 2063871 PMC1683227

[B15] Ikeda Y Dalton JC Day JW Ranum LPW Pagon RA, Adam MP, Bird TD, Dolan CR, Fong CT, Stephens K. Seattle WA Spinocerebellar Ataxia Type 8 GeneReviews 2001 University of Washington, Seattle

[B16] Nozaki K Onodera O Takano H Tsuji S Amino acid sequences flanking polyglutamine stretches influence their potential for aggregate formation Neuroreport 2001 12 15 3357 3364 10.1097/00001756-200110290-00042 11711886

[B17] Pulst SM Santos N Wang D Yang H Huynh D Velazquez L Figueroa KP Spinocerebellar ataxia type 2: polyQ repeat variation in the CACNA1A calcium channel modifies age of onset Brain : a journal of neurology 2005 128 Pt 10 2297 2303 16000334 10.1093/brain/awh586

[B18] Finke JM Cheung MS Onuchic JN A structural model of polyglutamine determined from a host-guest method combining experiments and landscape theory Biophysical journal 2004 87 3 1900 1918 10.1529/biophysj.104.041533 15345567 PMC1304594

[B19] Li X Li H Li X-J Intracellular degradation of misfolded proteins in polyglutamine neurodegenerative diseases Brain Research Reviews 2008 59 1 245 252 10.1016/j.brainresrev.2008.08.003 18773920 PMC2577582

[B20] Cote S Wei G Mousseau N All-atom stability and oligomerization simulations of polyglutamine nanotubes with and without the 17-amino-acid N-terminal fragment of the Huntingtin protein The journal of physical chemistry B 2012 116 40 12168 12179 10.1021/jp306661c 22978784

[B21] Kubota H Kitamura A Nagata K Analyzing the aggregation of polyglutamine-expansion proteins and its modulation by molecular chaperones Methods 2011 53 3 267 274 10.1016/j.ymeth.2010.12.035 21195182

[B22] Perney NM Braddick L Jurna M Garbacik ET Offerhaus HL Serpell LC Blanch E Holden-Dye L Brocklesby WS Melvin T Polyglutamine aggregate structure in vitro and in vivo; new avenues for coherent anti-Stokes Raman scattering microscopy PloS one 2012 7 7 e40536 10.1371/journal.pone.0040536 22911702 PMC3401212

[B23] Wang Y Voth GA Molecular dynamics simulations of polyglutamine aggregation using solvent-free multiscale coarse-grained models The journal of physical chemistry B 2010 114 26 8735 8743 10.1021/jp1007768 20550147

[B24] Lakhani VV Ding F Dokholyan NV Polyglutamine induced misfolding of huntingtin exon1 is modulated by the flanking sequences PLoS computational biology 2010 6 4 e1000772 10.1371/journal.pcbi.1000772 20442863 PMC2861695

[B25] Kawaguchi Y Okamoto T Taniwaki M Aizawa M Inoue M Katayama S Kawakami H Nakamura S Nishimura M Akiguchi I CAG expansions in a novel gene for Machado-Joseph disease at chromosome 14q32.1 Nature genetics 1994 8 3 221 228 10.1038/ng1194-221 7874163

[B26] Imbert G Saudou F Yvert G Devys D Trottier Y Garnier JM Weber C Mandel JL Cancel G Abbas N Cloning of the gene for spinocerebellar ataxia 2 reveals a locus with high sensitivity to expanded CAG/glutamine repeats Nature genetics 1996 14 3 285 291 10.1038/ng1196-285 8896557

[B27] Miller J Rutenber E Muchowski PJ Polyglutamine dances the conformational cha-cha-cha Structure 2009 17 9 London, England : 1993 1151 1153 10.1016/j.str.2009.08.004 19748335 PMC2830152

[B28] Kim MW Chelliah Y Kim SW Otwinowski Z Bezprozvanny I Secondary structure of Huntingtin amino-terminal region Structure 2009 17 9 London, England : 1993 1205 1212 10.1016/j.str.2009.08.002 19748341 PMC2863341

[B29] Kim M Beta conformation of polyglutamine track revealed by a crystal structure of Huntingtin N-terminal region with insertion of three histidine residues Prion 2013 7 3 23370273 10.4161/pri.23807 PMC3783107

[B30] Esposito L Paladino A Pedone C Vitagliano L Insights into structure, stability, and toxicity of monomeric and aggregated polyglutamine models from molecular dynamics simulations Biophysical journal 2008 94 10 4031 4040 10.1529/biophysj.107.118935 18234827 PMC2367175

[B31] Miettinen MS Knecht V Monticelli L Ignatova Z Assessing polyglutamine conformation in the nucleating event by molecular dynamics simulations The journal of physical chemistry B 2012 22770401 10.1021/jp305065c

[B32] Runthala A Protein structure prediction: challenging targets for CASP10 Journal of biomolecular structure & dynamics 2012 30 5 607 615 10.1080/07391102.2012.687526 22731875

[B33] Moult J Fidelis K Kryshtafovych A Rost B Tramontano A Critical assessment of methods of protein structure prediction - Round VIII Proteins 2009 77 Suppl 9 1 4 19774620 10.1002/prot.22589

[B34] Zhang Y I-TASSER server for protein 3D structure prediction BMC bioinformatics 2008 9 40 10.1186/1471-2105-9-40 18215316 PMC2245901

[B35] Crooks GE Hon G Chandonia JM Brenner SE WebLogo: a sequence logo generator Genome research 2004 14 6 1188 1190 10.1101/gr.849004 15173120 PMC419797

[B36] Zhang Y Skolnick J TM-align: a protein structure alignment algorithm based on the TM-score Nucleic acids research 2005 33 7 2302 2309 10.1093/nar/gki524 15849316 PMC1084323

[B37] Zhang Y Skolnick J Scoring function for automated assessment of protein structure template quality Proteins 2004 57 4 702 710 10.1002/prot.20264 15476259

[B38] Slater AW Castellanos JI Sippl MJ Melo F Towards the development of standardized methods for comparison, ranking and evaluation of structure alignments Bioinformatics (Oxford, England) 2013 29 1 47 53 10.1093/bioinformatics/bts600 23060612

[B39] Berman HM Westbrook J Feng Z Gilliland G Bhat TN Weissig H Shindyalov IN Bourne PE The Protein Data Bank Nucleic acids research 2000 28 1 235 242 10.1093/nar/28.1.235 10592235 PMC102472

[B40] Raman S Vernon R Thompson J Tyka M Sadreyev R Pei J Kim D Kellogg E DiMaio F Lange O Structure prediction for CASP8 with all-atom refinement using Rosetta Proteins 2009 77 Suppl 9 89 99 19701941 10.1002/prot.22540 PMC3688471

[B41] Roy A Kucukural A Zhang Y I-TASSER: a unified platform for automated protein structure and function prediction Nat Protocols 2010 5 4 725 738 10.1038/nprot.2010.5 PMC2849174 20360767

[B42] Leaver-Fay A Tyka M Lewis SM Lange OF Thompson J Jacak R Kaufman K Renfrew PD Smith CA Sheffler W ROSETTA3: an object-oriented software suite for the simulation and design of macromolecules Methods in enzymology 2011 487 545 574 21187238 10.1016/B978-0-12-381270-4.00019-6 PMC4083816

[B43] The PSIPRED protein sequence analysis workbench http://bioinf.cs.ucl.ac.uk/psipred/

[B44] Jufo9D Server http://www.meilerlab.org/index.php/servers/show?s_id = 5

[B45] Karplus K SAM-T08, HMM-based protein structure prediction Nucleic acids research 2009 37 Web Server W492 497 10.1093/nar/gkp403 19483096 PMC2703928

[B46] Gront D Kulp DW Vernon RM Strauss CE Baker D Generalized fragment picking in Rosetta: design, protocols and applications PloS one 2011 6 8 e23294 10.1371/journal.pone.0023294 21887241 PMC3160850

[B47] Kabsch W Sander C Dictionary of protein secondary structure: pattern recognition of hydrogen-bonded and geometrical features Biopolymers 1983 22 12 2577 2637 10.1002/bip.360221211 6667333

[B48] Pettersen EF Goddard TD Huang CC Couch GS Greenblatt DM Meng EC Ferrin TE UCSF Chimera--a visualization system for exploratory research and analysis Journal of computational chemistry 2004 25 13 1605 1612 10.1002/jcc.20084 15264254

[B49] R Development Core Team: R: A language and environment for statistical computing Vienna, Austria: R Foundation for Statistical Computing 2011

